# Methanol poisoning in the emergency department: methanol blood levels, prognosis, and sequelae outcomes

**DOI:** 10.1007/s11845-025-03982-9

**Published:** 2025-06-21

**Authors:** Kürşat Sarıbaş, Ayça Açıkalın Akpınar, Pınar Efeoğlu Özşeker, Ömer Taşkın, Nezihat Rana Dişel, Gül Filiz Devecioğlu

**Affiliations:** 1 Department of Emergency Medicine, Çukurova University, Adana, Turkey; 2 Department of Forensics Medicine, Çukurova University, Adana, Turkey

**Keywords:** Metabolic acidosis, Methanol, Poisoning, Toxic alcohol

## Abstract

**Background:**

Methanol poisoning is a critical condition marked by severe metabolic acidosis, shock, and organ failure, often leading to high morbidity, mortality, and sequelae such as permanent blindness.

**Aims:**

This study retrospectively analyzes methanol poisoning cases to provide insights into diagnosis, treatment, outcomes, and sequelae rates.

**Methods:**

Patients diagnosed with methanol poisoning in a university-based emergency department between 2015 and 2023 were analyzed. Data on demographics, presenting complaints, diagnostics, treatments, hospitalization, outcomes, and sequelae were collected.

**Results:**

Among 116 patients, the mean age was 48.3 ± 13.5 years, and 94.8% (*n* = 110) were male. Alcohol poisoning accounted for 110 cases, while six involved other substances. Eighty-nine patients were discharged, and 27 died. Ocular findings predominated among survivors, while altered consciousness was the most common complaint in deceased patients. Deceased patients exhibited significantly lower pH and HCO_3_ levels and higher base deficit, anion gap, and lactate levels. Blood methanol levels (mean, 64.5 ± 79.9 mg/dL) showed no correlation with mortality. Treatments included hemodialysis (89.7%), hemofiltration (19.8%), ethyl alcohol (77.6%), fomepizole (15%), and NaHCO_3_ (78.1%). Visual sequelae developed in 33.7% of patients, while neurologic sequelae occurred in 6.2%.

**Conclusion:**

Methanol poisoning remains a severe clinical issue with high morbidity and mortality. Low pH, HCO_3_, and high anion/lactate levels are poor prognostic indicators. Early treatment improves outcomes, yet disabling visual sequelae are frequent. Developing rapid diagnostic kits for methanol and formic acid detection is essential for improving early diagnosis and management.

## Introduction

Toxic alcohol poisoning is defined as poisoning that occurs after accidental or intentional ingestion of methanol, ethylene glycol, and isopropanol, which are called toxic alcohols [1]. Unfortunately, toxic alcohol poisoning continues to be a serious global health concern, with the potential to result in death and permanent sequelae. A thorough analysis of cases reveals that methanol poisoning is the most prevalent type in Türkiye. Methanol (CH_3_OH), also known as methyl alcohol or wood alcohol, is the simplest known aliphatic alcohol and is a solvent that is widely used in industry. Regrettably, the illegal use of methanol has become a prevalent practice, particularly in the production of counterfeit alcoholic drink, with the intent to reduce production costs. Consumers of such illicit spirits are consequently subjected to the risk of methanol poisoning (MP) [2].

The diagnosis of MP in the emergency department (ED) is important due to the high morbidity and mortality associated with the condition specially when anamnesis is not available. However, the diagnosis of MP by the ED physician is challenging. Some patients may conceal the substance they have consumed due to social concerns, while others may be unaware of their ingestion due to the presence of counterfeit alcoholic drink producers. The inability to elicit a detailed anamnesis from patients, particularly in cases of alcohol intoxication, can hinder the diagnostic process, as symptoms such as blurred vision, altered mental state, and impaired consciousness may not be readily apparent [3]. At the same time, emergency measurement of blood levels of methanol and its metabolites is not available in almost any ED. Victims and relatives of victims confuse intoxication with drunkenness and have difficulty in coming to the hospital due to intoxication, and other social reasons lead to late diagnosis of intoxication.

Methanol poisoning may present with symptoms similar to ethanol ingestion, such as fatigue, nausea, headache, and vomiting, but may also present with severe symptoms such as blurred vision, loss of vision, chest pain, respiratory distress, coma, and convulsions. Methanol is mostly converted to formic acid through hepatic metabolism. Formic acid, a metabolite of methanol, is the main metabolite responsible for metabolic acidosis, blindness, coma, central nervous system (CNS) pathologies, and death. Hemodialysis is recommended in all cases of MP with visual impairment and signs of end-organ failure. In order to treat the patient effectively, immediate access to resources such as hemodialysis is the keystone of treatment [2, 3].

The objective of this study was to analyze MP patients and to provide the literature with data on diagnosis, treatment, antidote use, hemodialysis access, patient outcome, and permanent sequelae rates. It is crucial to understand the global and regional challenges in the diagnosis and treatment of MP, including the identification of significant parameters for early diagnosis, permanent sequelae, and mortality rates. This knowledge is essential for the development of effective treatment and preventive measures.

## Material and method

This retrospective, descriptive study was conducted in the Adult ED of a university hospital between January 1, 2015, and June 1, 2023, with approval from the local university’s ethics committee (date December 8, 2023, decision number 6). The term “toxic alcohols” refers to substances such as methanol, isopropyl alcohol, and ethylene glycol. The hospital is located in Adana province in Turkey. The region’s mild climate is characterized by minimal use of antifreeze. Notably, the region experiences a high prevalence of counterfeit alcoholic drink ingestions, with patients typically evaluated for MP upon examination of the substance that caused the exposure.

A retrospective analysis was conducted on patients with MP, utilizing ED examination cards and electronic patient records. The demographic data collected included patient age and gender at the time of presentation, medical history, complaints at the time of presentation, laboratory results (including venous blood gas and methanol and ethanol levels at presentation to the our ED) and findings of the imaging studies, antidotes administered in the ED, treatment modalities, length of hospital stay, sequelae, and ED outcomes.

The sequelae status of the patients included in the study was obtained by reviewing the data in the patient files. When necessary, the patients were contacted by telephone to determine their sequelae status. Patients were grouped as survived and deceased patients and analyzed.

### Statistical analysis

The data obtained in the study were analyzed in SPSS IBM Statistics version 22. The mean and standard deviation (SD) were employed to represent quantitative data, while the number of cases (*n*) and percentages (%) were utilized to represent qualitative data. The Kolmogorov–Smirnov test was employed to assess the distribution of quantitative data. The Mann–Whitney *U* test was employed to analyze nonparametric data, while the Student *t*-test was utilized to analyze parametric data. The chi-square test was employed to analyze the qualitative data. For the study, a *p*-value less than 0.05 was considered to be statistically significant.

## Results

A total of 116 patients whose data were available were included in the study. The mean age of these patients was 48.3 ± 13.5 years, and 94.8% (*n* = 110) were male. It was determined that 96 (82.7%) of the patients were poisoned with counterfeit alcoholic drink and 6 (5.2%) with cologne and/or spirits. A total of 27 (23.2%) patients died, one of whom died in the ED before being admitted to the intensive care unit, and 26 patients deceased in the intensive care unit. The median age of the deceased patients was 46 (37.5–63) years and that of the living patients was 51 (40–57) years. There was no statistically significant difference between the ages and mortality rates of the patients (*p* = 0.868). Table 1 shows the demographic characteristics, substance ingested, background characteristics, and outcomes of the patients.

**Table 1:** Characteristics of the patients

Age (years), mean ± SD (min–max)		48.3 ± 13.5 (19-82)
Gender, *n* (%)	Male	110 (94.8)
	Female	6 (5.2)
Substance taken, *n* (%)	Counterfeit alcoholic drink	110 (94.8)
	Cologne/spirits	6 (5.2)
Disease in medical history, *n* (%)	Hypertension	17 (14.7)
	Psychiatric disorder	11 (9.5)
	Diabetes mellitus	8 (6.9)
	Coronary artery disease	8 (6.9)
	Drug/substance abuse	4 (3.4)
	COPD/asthma	3 (2.6)
	Malignancy	2 (1.7)
	Other	8 (6.9)
Outcome, *n* (%)	Exitus	27 (23.2)
	Discharged	89 (67.8)

*SD* standard deviation, *COPD* chronic obstructive pulmonary disease

The analysis of the entire patient cohort revealed that the most prevalent presenting complaint was blurred vision. In the present study, blurred vision was the most prevalent presenting complaint among survived patients, while impaired consciousness was the most common complaint among deceased patients. It was determined that methanol levels in the blood taken at presentation were not different between patients who presented with blurred vision and those who presented with total blindness (*p* values *p* = 0.754 and *p* = 0.806, respectively). Furthermore, a comparative analysis revealed that headache and shortness of breath were more prevalent among deceased patients than among survived patients. The findings are delineated in Table 2.

**Table 2:** Patients’ complaints at presentation

	All patients	Outcome		*p*
		Deceased	Survived	
Blurred vision, *n* (%)	76 (65.5)	14 (51.9)	62 (69.7)	0.088^a^
One eye, *n* (%)	4 (5.3)	0 (0.0)	4 (5.3)	n/a
Both eyes, *n* (%)	72 (94.7)	14 (18.4)	58 (76.3)	n/a
Impaired consciousness,* n* (%)	51 (44)	23 (85.2)	28 (31.5)	< 0.001^a^
Nausea/vomiting,* n* (%)	47 (40.5)	15 (55.6)	32 (36)	0.069^a^
Shortness of breath, *n* (%)	36 (31)	18 (66.7)	18 (20.2)	< 0.001^a^
Headache, *n* (%)	27 (23.3)	11 (40.7)	16 (18)	0.014^a^
Visual loss, *n* (%)	10 (8.6)	4 (14.8)	6 (6,7)	0.239^b^
One eye, *n* (%)	0 (0.0)	0 (0.0)	0 (0.0)	n/a
Both eyes, *n* (%)	10 (100)	4 (40)	6 (60)	n/a
Chest pain, *n* (%)	12 (10.3)	3 (11.1)	9 (10.1)	0.881^a^

^a^Pearson’s chi-square test

^b^Fischer’s exact test

Seven of the patients included in the study were intubated from an external center and a total of 21 patients required intubation and mechanical ventilation in the ED. The findings are presented in Table 3.

**Table 3:** Patients’ vital signs at the time of presentation

	All patients	Outcome		*p*
		Deceased	Survived	
Systolic blood pressure (mmHg), *mean* ± *SD*	116.6 ± 28.6	91.5 ± 26.1	124.2 ± 24.8	< 0.001^b^
Diastolic blood pressure (mmHg), *mean* ± *SD*	69.8 ± 15.8	55.4 ± 15.2	74.1 ± 13.3	< 0.001^b^
Heart rate (bpm), *mean* ± *SD*	92.4 ± 21.7	103.2 ± 31.5	89.1 ± 16.5	0.002^b^
Respiratory rate (bpm), *mean* ± *SD*	20.3 ± 6.6	24.4 ± 10.5	19.2 ± 4.6	0.025^b^
O_2_ saturation (%), *mean* ± *SD*	95.4 ± 13.4	91.7 ± 20.2	96.4 ± 10.7	0.003^b^
Body temperature (°C), *mean* ± *SD*	37 ± 5.8	38.6 ± 11.9	36.5 ± 0.3	0.208^b^

^b^Mann-Whitney *U* test

The laboratory values of the patients are presented in Table 4, which demonstrates that there was no statistically significant difference in methanol and ethyl alcohol levels between the survived and deceased patients.

**Table 4:** Patients’ laboratory results

	All patients	Outcome		*p*
		Deceased	Survived	
Glucose (mg/dL) *mean* ± *SD*	160.1 ± 87.5	201.7 ± 97.2	147.5 ± 80.8	0.004^b^
BUN (mg/dL), *mean* ± *SD*	14.4 ± 13.4	16.2 ± 7.1	13.9 ± 14.8	0.014^b^
Creatinine (mg/dL), *mean* ± *SD*	1.1 ± 0.6	1.7 ± 0.6	0.9 ± 0.4	< 0.001^b^
Total bilirubin (mg/dL), *mean* ± *SD*	0.8 ± 1	1.2 ± 1.9	0.7 ± 0.5	0.288^b^
Direct bilirubin (mg/dL), *mean* ± *SD*	0.3 ± 0.5	0.5 ± 0.9	0.2 ± 0.1	0.033^b^
Lactate dehydrogenase (U/L), *mean* ± *SD*	255.4 ± 217	423.9 ± 355	204.2 ± 113.9	< 0.001^b^
Aspartate aminotransferase (U/L), *mean* ± *SD*	64.4 ± 64.9	121.9 ± 104.6	47 ± 30.8	< 0.001^b^
Alanine aminotransferase (U/L), *mean* ± *SD*	41.7 ± 35.2	67.1 ± 46	34 ± 27.1	< 0.001^b^
Sodium (mmol/L), *mean* ± *SD*	137.2 ± 5.4	139.5 ± 6	136.5 ± 5.1	0.013^b^
Potassium (mmol/L), *mean* ± *SD*	4.7 ± 1.2	5.4 ± 1.5	4.4 ± 1	0.004^b^
Chlorine (mmol/L), *mean* ± *SD*	101.7 ± 7	100.5 ± 4.4	102.1 ± 7.7	0.023^b^
Amylase (U/L), *mean* ± *SD*	151.7 ± 24.5	236 ± 23.7	126.2 ± 101.9	< 0.001^b^
C-Reactive protein (mg/dL), *mean* ± *SD*	11.1 ± 43.7	33.3 ± 86.9	4.3 ± 8.4	< 0.001^b^
pH, *mean* ± *SD*	7.01 ± 0.2	6.8 ± 0.2	7.2 ± 0.1	< 0.001^b^
HCO_3_ (mEq/L), *mean* ± *SD*	12.9 ± 19.6	6.8 ± 2.5	12.5 ± 5.5	< 0.001^b^
Base deficit, *mean* ± *SD*	− 16.3 ± 8.6	− 25.2 ± 6.1	− 13.6 ± 7.3	< 0.001^b^
Anion gap, *mean* ± *SD*	25.4 ± 9	32.9 ± 7	23.1 ± 8.4	< 0.001^b^
White blood cell count (× 10^3^/µL), *mean* ± *SD*	12 ± 5.5	15.3 ± 5.7	10.9 ± 5	< 0.001^b^
Hematocrit (%), *mean* ± *SD*	44.5 ± 6.7	43.9 ± 7	44.7 ± 6.7	0.589^c^
Platelet count (× 10^3^/µL), *mean* ± *SD*	238.2 ± 90.4	245 ± 105.6	236.1 ± 85.8	0.657^c^
Methanol (mg/dL), *mean* ± *SD*	64.5 ± 79.9	86.2.8 ± 75.4 (*n* = 20)	57.8 ± 80.6 (*n* = 64)	0.055^b^
Ethyl alcohol (mg/dL), *mean* ± *SD*	18.2 ± 17.1	2.5 (*n* = 1)	21.3 ± 17 (*n* = 5)	0.333^b^

*BUN* blood urea nitrogen, *SD* standard deviation

^b^Mann-Whitney *U* test

^c^Student’s *t*-test

The most common diagnostic procedure performed on patients was brain CT imaging. An evaluation of the brain CT scans of patients who were excluded revealed that the frequency of cerebral edema (*p* = 0.001) and intracranial hematoma was significantly higher in those who did not survive. Furthermore, diffusion magnetic resonance imaging (MRI) findings revealed diffusion limitation signal in 12 out of 13 patients (92.3%) who underwent diffusion MRI. No significant difference was observed between survived and deceased patients. The findings are outlined in Table 5.

**Table 5:** Patients’ imaging findings

	All patients	Outcome		*p*
		Deceased	Survived	
Brain CT imaging, *n* (%)	50 (42.2)	15 (55.6)	35 (39.3)	0.136^a^
No features	31 (38)	5 (33.3)	26 (74.1)	0.006^a^
Brain edema	17 (34)	10 (66.7)	7 (20)	0.001^a^
Cerebral ischemia	14 (28)	7 (46.7)	7 (20)	0.054^a^
Intracranial hemorrhage	4 (8)	3 (20)	1 (2.9)	0.041^a^
Brain MRI, *n* (%)	23 (19.8)	8 (29.6)	15 (16.9)	0.107^a^
No features	4 (17.4)	0	4 (26.7)	n/a
Brain edema	13 (56.5)	7 (87.5)	6 (40)	0.029^a^
Cerebral ischemia	17 (73.9)	7 (87.5)	10 (66.7)	0.278^a^
Intracranial hemorrhage	2 (8.7)	1 (12.5)	1 (6.7)	0.636^a^
Brain diffusion MRI, *n* (%)	13 (11.2)	5 (18.5)	8 (9)	0.169^a^
No features	1 (7.7)	0	1 (12.5)	n/a
Diffusion limitation signal	12 (92.3)	5 (100)	7 (87.5)	

^a^Pearson’s chi-square test

^b^Mann-Whitney *U* test

An analysis of the treatments administered to patients in ED revealed that 104 (89.7%) of 116 patients received hemodialysis, while 23 (19.8%) received hemofiltration. The mean duration of hemodialysis was 5.4 ± 2.6 h. The mean duration of hospitalization was 5.8 ± 5.5 days. The findings are presented in Table 6.

**Table 6:** Treatments applied and length of hospital stay

		All patients	Outcome		*p*
			Deceased	Survived	
Treatment applied, *n* (%)	Hemodialysis	104 (89.7)	23 (85.2)	81 (91)	0.384^a^
	Hemofiltration	23 (19.8)	6 (22.2)	17 (19.1)	0.722^a^
	Ethyl alcohol	90 (77.6)	19 (70.4)	71 (79.8)	0.305^a^
	Fomepizole	17 (15)	4 (14.8)	13 (15.1)	0.970^a^
	NaCHO_3_	89 (78.1)	27 (100)	62 (71.3)	0.002^a^
	Folic acid	58 (51.8)	10 (37)	48 (56.5)	0.078^a^
Hospitalization days, *mean ± SD*		5.8 ± 5.5	8 ± 7.7	5.2 ± 4.51	0.353^b^

^a^Pearson’s chi-square test

^b^Mann-Whitney *U* test

Visual sequelae were observed in 33.7% (*n* = 30) and neurologic sequelae in 6.2% (*n* = 7) of the patients.

## Discussion

Toxic alcohol poisoning continues to be a leading cause of mortality. In certain regions, it has been observed to periodically trigger toxic disasters, particularly due to its use in the production of counterfeit alcoholic drink [1]. The confluence of factors, including the challenges in accessing alcohol products due to the pandemic, the subsequent unemployment, and the societal repercussions of economic crises in various countries, has precipitated an escalation in alcohol prices within our nation, thereby inciting an augmentation in the production of counterfeit alcoholic drink [4]. At the same time, the fact that methanol is cheaper than ethyl alcohol has led counterfeit alcoholic drink producers to use methanol. Therefore, there has been an increase in the frequency of patients with MP [4, 5]. Furthermore, toxic alcohols such as methanol, ethylene glycol, and isopropyl alcohol are commonly used in a variety of hardware and household products. Consequently, exposure to these alcohols, whether accidental or suicidal, can result in toxic alcohol poisoning [6]. An evaluation of the toxic alcohols ingested by the patients included in this study revealed that all cases were methanol. A global analysis of toxic alcohol poisoning cases reveals that approximately 20% of cases are attributed to ethylene glycol. Given the temperate climate of our nation, particularly in our region, the utilization of antifreeze is minimal. Consequently, cases of accidental or suicidal antifreeze exposure are rarely reported. All cases were found to be instances of methanol exposure.

In assessing alcohol intake in terms of gender in Türkiye, it is evident that the rate of alcohol use in men is significantly higher than in women. Moreover, it has been documented that the male demographic is significantly overrepresented among workers in sectors that utilize toxic alcohol. Gülen et al. reported that 95.5% of the patients were male, with a mean age of 48.41 ± 13.12 years among males and a mean age of 34.33 ± 8.96 years among females [7]. Sadeghi et al. reported that the majority of patients were male (88.1%), and the mean age was 41.5 years [8]. Similar to other studies, this present research indicates that males constituted the vast majority of the exposed population (94.8%), with an average age of 48.3 ± 13.5 years. This phenomenon has been linked to increased alcohol consumption, particularly among adult males. Indeed, adult males with a higher alcohol consumption have been shown to encounter counterfeit alcoholic drink with greater frequency.

Literature on the subject indicates that accidental exposure to methanol is a prevalent occurrence. In the present study, the majority of patients exhibited accidental exposure to methanol. A comprehensive review of the extant literature reveals that Kaewput et al. reported a 56% prevalence of accidental exposure to methanol among patients admitted with MP [9]. Eke et al. reported that the sources of methanol in 22 cases were spirits in 3 cases, cologne in 10 cases, and spirits and cologne in 1 case, and no information about the source could be reached in 8 cases [10]. The present study determined that the source of toxic alcohol was counterfeit alcoholic drink in 82.7% of cases, and 5.2% of cases involved exposure to MP with other substances (spirits, cologne, etc.). This phenomenon is believed to be associated with the rise in the production and consumption of counterfeit alcoholic drink, which has been triggered by the substantial increase in alcohol prices within Türkiye. The study also noted that some individuals with alcohol use disorder may have resorted to consuming spirits or cologne due to financial constraints, while others may have unintentionally consumed these substances.

In their study, Hovda et al. stated that the most common presenting complaints were visual impairment (55%) and dyspnea (41%) [11]. As indicated by the study conducted by Sadeghi et al., the most prevalent clinical grievance was visual disturbances, constituting 75.25% of the cases. This was followed by nervous, gastrointestinal, and respiratory disorders in that order [8]. Cömertpay et al. stated that the most common reason for presentation was visual impairment (70.6%), followed by nausea and vomiting (35.3%) and shortness of breath (35.3%), and that there was no difference in terms of the complaint of presentation in patients survived and deceased [12]. As indicated in the study by Gülen et al., 82.4% of patients with gastrointestinal symptoms were discharged without sequelae, while 17.6% were discharged with sequelae. Furthermore, 57.1% of patients with visual impairment were discharged without sequelae, while 26.2% were discharged with sequelae. It is important to note that 88.2% of patients with respiratory depression and 58.1% of patients with altered consciousness were deceased [7]. In the present study, the most common complaints of patients admitted to the ED were blurred vision. The frequency of disturbance of consciousness, apneic breathing, shortness of breath, and headache was higher in deceased patients compared to living patients. This observation is attributed to the fact that individuals seek medical attention at the ED primarily due to the most bothersome symptoms, namely blurred vision and nausea/vomiting. Furthermore, the association between blurred vision, which arises subsequent to alcohol consumption in community settings, and methanol intake, a known cause of blindness, prompts individuals experiencing this condition to promptly seek medical attention at the EDs. The prevalence of impaired consciousness is also of concern, particularly given the referral of patients with critical conditions from peripheral hospitals to our institution. This study revealed that patients with blurred vision exhibited a higher prevalence of this condition. This suggests that these patients may have been more likely to receive early diagnosis and treatment by promptly seeking care at the ED, potentially avoiding the exacerbation of their condition.

When the relationship between vital measurements at the time of presentation and prognosis was evaluated, Arslan et al. reported that blood pressure was lower in the deceased patients; however, they did not find a relationship between mortality and heart rate [13]. Duyan et al. stated that diastolic blood pressure and saturation were lower in the deceased patients; systolic blood pressure, heart rate, and fever were similar in living and exitus patients [14]. In the present study, systolic and diastolic blood pressure and saturation values were found to be significantly lower, and pulse and respiratory rates were found to be significantly higher in patients who died. It is hypothesized that the divergent outcomes observed in studies conducted in disparate medical centers are attributable to the duration of hospital admission, which is directly proportional to the increase in formic acid, a toxic metabolite.

In the study conducted by Gülen et al., an evaluation was made of the relationship between the laboratory results of patients with methanol intoxication and mortality. The study found that white blood cell, glucose, blood urea nitrogen (BUN), creatinine, and aspartate aminotransferase (AST) values were higher, and hemoglobin values were lower in patients who died [7]. Cömertpay et al. reported that glucose, urea, and white blood cell levels of exitus and living patients were similar; creatinine value was higher in exitus patients [12]. In the present study, a comprehensive evaluation was conducted to determine the effects of MP on various biological parameters. The investigation revealed that several key indicators, including white blood cell count, glucose, BUN, creatinine, direct bilirubin, lactate dehydrogenase (LDH), AST, ALT, sodium, potassium, amylase, and C-reactive protein (CRP), exhibited significant elevations. Concurrently, the chlorine level was observed to undergo a substantial decrease. It is hypothesized that acute phase reactants, such as white blood cell count and CRP, and blood glucose levels increase as a result of inflammation and increased stress hormones in the body due to MP. It is further postulated that renal and liver function tests are significantly affected, particularly in the deceased patients, as elevated levels of toxic metabolites can lead to shock and multi-organ damage. A notable consideration is the potential for patients to exhibit chronic liver toxicity resulting from prolonged alcohol consumption, which further complicates the management of these cases.

In the later stages of MP, formic acid has been observed to inhibit mitochondrial cytochrome-c oxidase, which represents the final step in the mitochondrial electron transport chain, thereby suppressing cellular respiration. Consequently, the Na^+^/K^+^ ATPase pump is inhibited due to a deficiency in ATP. This sequence of events leads to metabolic acidosis and an accumulation of lactate [1]. Liu et al. accepted pH below 7 as the lethal limit value in patients with MP, while Roberts et al. defined the lethal pH range as 6.64–7.29 [15, 16]. In their study, Hovda et al. stated that the pH median of the patients was 7.2, the HCO_3_ median was 6 mmol/L, the base deficit was 22 mmol/L, and the anion gap was 25 meq/L; patients who were excluded or recovered with sequelae had deeper acidosis and higher anion and base deficits [11]. In the present study, the findings were consistent with the literature. Specifically, the pH and HCO_3_ levels were found to be significantly lower, while the base deficit, anion gap, and lactate levels were found to be significantly higher in the deceased patients. The hypothesis that elevated formic acid levels impede mitochondrial cytochrome-c oxidase function, inducing cellular hypoxia leading to acidosis and lactate accumulation, is postulated. This hypothesis is further supported by the observation that impaired pumps, resulting from energy deficiency, contribute to anion gap and base deficit exacerbation, thereby worsening the aforementioned condition.

In patients with MP, the blood methanol level can be measured, particularly in forensic toxicology laboratories, where the results are typically obtained after a significant delay. Today, the measurement of methanol or formic acid is not feasible at the bedside or in most emergency laboratories in hospitals. Emergency diagnosis is predominantly reliant on anamnesis and ancillary tests, excluding those specific to methanol and formic acid, such as blood gas and biochemical measurements. This limitation can result in delays in diagnosis, particularly in cases where anamnesis is not feasible. A review of the literature reveals that Tuncez et al. reported that methanol levels were found to be very low in 36.4% of the cases. In a study focusing on blood methanol level analysis, the concentration range was reported to be between 0 and 642 mg/dL, with a mean concentration of 178 mg/dL [17]. In their study, Rulisek et al. found a methanol concentration of 29 mmol/L in deceased patients and 21 mmol/L in living patients, concluding that there was no difference between the groups [18]. In another study, it was reported that there was no relationship between blood methanol levels and mortality [19]. In the present study, the blood methanol levels of 84 patients were examined in a forensic toxicology laboratory setting. The mean blood methanol level was determined to be 64.5 ± 79.9 mg/dL. While methanol levels were observed to be higher in cases resulting in mortality, this variation did not attain statistical significance. Given that formic acid is the predominant clinical determinant in MP and the conversion rate remains unknown, it is concluded that no relationship exists between methanol level and clinical outcome. We determined that methanol blood level is not a guiding factor in the treatment and follow-up processes of patients. Nevertheless, the decision for hemodialysis in patients with methanol poisoning can be based on methanol blood level. However, the laboratory analysis that is decisive in the management of patients should be formic acid instead of methanol. Clinical and laboratory markers should be prioritized over methanol blood level when making hemodialysis decisions.

It has been stated that imaging plays a critical role in cases of MP, with CT showing signs of bilateral hemorrhagic putaminal necrosis, hypodense areas in the brain, and cerebral and intraventricular hemorrhage. The most characteristic MRI finding in MP has been reported to be bilateral putaminal necrosis with varying degrees of concomitant hemorrhage [20]. In another study, it was suggested that hemorrhagic lesions were due to the direct toxic effect of formic acid rather than hemodialysis [21]. In another study by Zakharov et al., CT/MRI scans of 46 patients with MP were evaluated; 52% had abnormal brain findings, and the most common lesions were putamen necrosis (16 patients) and hemorrhage (15 patients) [22]. Vaneckova et al. examined the MRI findings of patients admitted to the hospital due to MP. The study revealed that patients with pathologic findings exhibited deeper acidosis and higher levels of methanol and formic acid compared to those without such findings. The study further noted that the diagnostic value of CT in the acute phase was low, with the ability to discern brain edema being the sole exception [23]. In the present study, lesions were detected in 62% of patients who underwent CT scans, with brain edema (34%) and cerebral ischemia (28%) being the most common. Intracranial hemorrhage developed in 8% (*n* = 4) of the patients, with no history of comorbidity or drug use. Among the 23 patients who underwent MRI, 82.6% exhibited lesions, with ischemia (73.9%) and brain edema (56.5%) being the most prevalent. The frequency of cerebral edema and hemorrhage on CT and cerebral edema on MRI was found to be high in patients who deceased. The etiology of brain lesions is believed to be multifactorial, involving direct damage, acidosis, hypoxia at the cellular level, and toxicity caused by MP and its metabolites. Furthermore, the direct toxicity of methanol and its metabolites and the intensive use of sodium bicarbonate, anticoagulants applied in dialysis, and other factors that disrupt coagulation (alcoholic cirrhosis, bleeding diathesis, etc.) have the potential to increase the frequency of intracranial hemorrhage in these patients. It is hypothesized that MRI is not used as a screening method in patients with methanol intoxication in our ED. MRI is performed for lesions that are thought to be CNS toxicity but not detected on CT, and therefore, the frequency of lesion detection on MRI is high.

The treatment of methanol toxicity can be approached in a number of ways. These include the provision of supportive care, the administration of antidotes such as fomepizole and ethanol, extracorporeal removal of toxic substances (dialysis), and the use of cofactor (folic acid) therapy [24]. In the study by Zakharov et al., ethanol and fomepizole were administered as antidotes to 68% and 23% of the patients, respectively, and the relationship between these antidotes (fomepizole and ethanol) and folate with mortality was compared. In this study, they found that antidotes did not affect the mortality rate, and patients who were started on folic acid had a lower rate of exitus [19]. Gülen et al. stated that ethanol, fomepizole, and folic acid treatment were given more frequently to survived patients compared to deceased patients; the frequency of NaHCO_3_ treatment was higher in deceased patients [7]. In the present study, ethanol was administered to 77.6% of the patients, while fomepizole was administered to 15%. While no relationship was found between antidote treatment and mortality, the frequency of NaHCO_3_ administration was found to be significantly higher in patients with a fatal outcome. Ethanol is presumably the preferred antidote due to its greater accessibility and cost-effectiveness compared to fomepizole. The increased administration of NaHCO_3_ in patients with more profound acidosis and lower HCO_3_ levels is a plausible explanation for this observation. Furthermore, the administration of methylprednisolone was observed in 33.6% of the patients in the study. The primary rationale for the high frequency of methylprednisolone administration to living patients is that eye consultation was performed in living patients with visual impairment, and methylprednisolone treatment was recommended to these patients by the eye clinic.

Hovda et al. reported that 73% of patients were dialyzed, and the median duration of dialysis was 6 h [11]. In a study, it was reported that 53.8% of dialyzed patients survived, 21.5% recovered with sequelae, and 24.6% were deceased. In this study, it was stated that there was no relationship between mortality and dialysis [7]. In the present study, the majority of patients (89.7%) underwent hemodialysis, while a smaller proportion (19.8%) underwent hemofiltration due to hemodynamic instability. The mean duration of dialysis was 5.4 ± 2.6 h, and no relationship was observed between hemodialysis or hemofiltration and mortality. The primary objectives of both hemodialysis and hemofiltration are the removal of toxic metabolites and the correction of electrolyte imbalance. However, it is important to note that the clinical and laboratory results of patients can serve not only as indications for hemodialysis but also as a basis for determining its duration.

Rulisek et al. reported that the hospitalization time of living patients was 32 h, while the hospitalization time of those who were excluded was 48 h [18]. A study was conducted to compare the duration of hospitalization for patients receiving either ethanol or fomepizole. The median length of hospitalization for the fomepizole group was found to be 6 days, while the median length of hospitalization for the ethanol group was 4 days. The analysis revealed that there was no significant difference between the two groups [25]. In this study, the mean length of hospitalization was 5.8 ± 5.5, and no significant difference was found between the groups in terms of length of hospitalization. The fact that the length of hospitalization in this study was longer than the literature is thought to be due to the fact that patients with ocular sequelae were transferred to the ophthalmology clinic, and the time spent in the ophthalmology service was included in the length of hospitalization in this study.

In many studies, the mortality rate due to MP has been reported differently. Rulisek et al. [18] reported a mortality rate of 26.7%; Ahmed et al. [26] reported a mortality rate of 66.7%; Sosnowska et al. [27] reported a mortality rate of 40%. The mortality rate observed in this study was 23.3%. The observed difference in mortality rates between this study and the existing literature can be attributed to several factors. Firstly, our hospital functions as a specialized toxicology center, which allows for more efficient management of patients with toxic substance poisoning. Secondly, the accessibility and duration of hemodialysis treatment at our facility is superior to that of many other centers. Thirdly, the medications available for treatment are consistently available, and our hospital team has extensive experience in managing MP.

When the sequelae rates of patients with methanol poisoning were evaluated, Sadeghi et al. reported that 16.8% of patients were discharged with sequelae [8]. In a study by Zakharov et al., it was reported that 7 (14%) of 50 patients were discharged with a diagnosis of visual sequelae, while 6 (12%) were discharged with both visual and CNS sequelae [25]. As a result of long-term follow-up of the patients, 40% long-term visual sequelae and 8% blindness were observed. In this study, 37 patients were discharged with sequelae (30 with visual sequelae and 7 with neurologic sequelae). Our rate of visual and neurologic sequelae is similar to the literature.

## Limitations

As the majority of the patient data were derived from hospital archives and automation records, a comprehensive analysis of alcohol consumption (methanol or ethanol), the duration of hospital admission, the onset of complaints, and the presence of other factors contributing to morbidity and mortality, particularly in unconscious patients, due to the inability to obtain detailed anamnesis, might not have been feasible. One of the most remarkable aspects of the study is that sequelae analysis was performed and methanol blood levels could be measured in the majority of patients. However, these levels are not decisive. Since formic acid level could not be measured in the toxicology laboratory of our center, an analysis could not be performed, but if it could have been, it could have a determinant effect on morbidity and mortality. Given that imaging procedures were not performed on patients deemed to have no indication, the diagnostic value of CT and MRI remains constrained.

## Conclusion

Methanol poisoning remains a major cause of morbidity and mortality, highlighting the importance of early diagnosis and prompt treatment. The finding that blurred vision after alcohol consumption is associated with lower mortality emphasizes its role as a critical early diagnostic sign. Given the ongoing risk of methanol intoxication in our region, increasing public awareness is crucial for early recognition and management. In patients presenting with hyperventilation, visual impairment, altered consciousness, and metabolic acidosis with an increased anion gap, methanol poisoning should be strongly suspected. Low pH and bicarbonate levels, together with elevated base deficit, anion gap, and lactate values, were observed more frequently among non-survivors and may be associated with worse clinical outcomes. Although early treatment can be effective, disabling visual sequelae remain common. The availability of point-of-care ethyl alcohol testing underlines the urgent need for rapid diagnostic kits for early methanol and late-stage formic acid detection, which could improve timely intervention and prognosis.

## Data Availability

Data is available upon request.
